# Inflection Point in Pressure Dependence of Ionic Conductivity as a Fingerprint of Local Structure Formation

**DOI:** 10.1021/acs.jpcb.3c08506

**Published:** 2024-05-08

**Authors:** S. Koymeth, B. Yao, M. Paluch, M. Dulski, M. Swadzba-Kwasny, Z. Wojnarowska

**Affiliations:** † Faculty of Science and Technology, Institute of Physics, University of Silesia in Katowice, 75 Pułku Piechoty 1A, Chorzów 41-500, Poland; ‡ Faculty of Science and Technology, Institute of Materials Science, University of Silesia in Katowice, 75 Pułku Piechoty 1A, Chorzów 41-500, Poland; § The QUILL Research Centre, School of Chemistry and Chemical Engineering, The Queen’s University of Belfast, David Keir Building, Stranmillis Road, Belfast BT9 5AG, U.K.

## Abstract

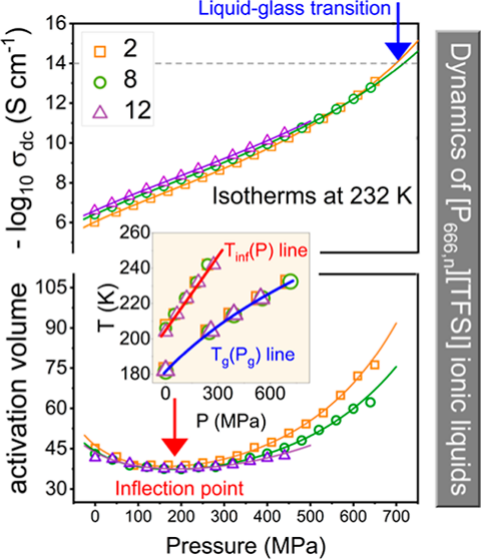

In this study, we employed dielectric spectroscopy to investigate the effect of temperature and pressure on the ion dynamics of phosphonium ionic liquids (ILs) differing by the length of an alkyl chain, [P_666,*n*_][TFSI] (*n* = 2, 6, 8, 12). We found that both temperature and pressure dependence of dc-conductivity (σ_dc_) determined for all examined ILs herein exhibit unique characteristics, unusual for aprotic ILs. Two regions differing by ion self-organization have been identified from the derivative analysis of σ_dc_(*T*^–1^) data. On the other hand, isothermal measurements performed at elevated pressure revealed a unique concave–convex character of σ_dc_(*P*) dependences, resulting in a clear minimum in the pressure behavior of activation volume. Such an inflection point characterizing the pressure dependence of σ_dc_ in [P_666,*n*_][TFSI] ILs can be considered an inherent feature of ion dynamics governed by structural self-assembly. Our results offer a unique perspective to link the ion mobility at various *T*–*P* conditions to the nanostructural organization of ionic systems.

## Introduction

Ionic liquids (ILs), which are liquid molten salts composed solely of ions, have become ubiquitous in the landscape of scientific research, spanning protein and DNA chemistry, organic solvents, catalysis, and energy storage.^1^ These applications demonstrate the versatility of ILs and their promising potential in various industries. The inherent feature of ILs is tailoring their properties, such as ionic conductivity, thermal stability, and vapor pressure, through the pairing of cations and anions.^2−4^ However, the physical arrangement of ions in bulk or at interfaces can also affect the mechanical and conducting properties of ILs.^5^ Therefore, a profound comprehension of self-organization in ILs is crucial to exploit their properties effectively and, thus, optimize their applications.

Growing evidence suggests that the tendency to ion self-assemble becomes prevalent among ILs.^5,6^ In 2013, it was suggested that ILs are the most promising class of amphiphile self-assembly media.^7^ Moreover, a robust correlation has been found between the structure of ILs and its amphiphile self-assembly ability, attributed to the spontaneous solvophobic segregation of charged and uncharged constituents into polar and nonpolar domains.^7−9^

Over the years, various experimental techniques have been used to characterize the nanostructure of ILs. For example, using X-ray diffraction, Triolo et al. provided the first empirical evidence of nanoscale heterogeneities in supercooled ILs.^10^ They found that these nanodomains are built up by neutral alkyl tails, which are surrounded by charges with a homogeneous spatial distribution governed by strong electrostatic interactions.^11^ On the other hand, nuclear magnetic resonance relaxation studies of various protic ILs revealed that the cation and anion can diffuse as an ion pair and tend to form hydrogen-bonded aggregates, especially when cation alkyl chains are longer than four carbons.^12,13^ Furthermore, Russina et al.^14^ emphasized the advantages of small and wide-angle X-ray scattering (SAXS/WAXS) in determining the size of structural heterogeneities in ILs. In addition, Raman,^15,16^ small-angle neutron scattering,^17^ rheology,^18^ infrared spectroscopy,^19^ and MD simulations^20,21^ are also effective and commonly utilized experimental methods for monitoring self-assembled nanostructures in ILs.

Broadband dielectric spectroscopy (BDS), usually employed for studies of the relaxation dynamics of glass-forming systems,^22,23^ can also be an effective tool for investigating the self-assembly of ILs. A slow, sub-α relaxation in the dielectric spectra attributed to polarization at the interfaces of polar and nonpolar domains of the aggregates has been found in a series of alkyl-imidazolium^24^ and phosphonium^25^ ILs. Furthermore, the temperature dependence of conductivity relaxation times (τ_σ_), describing the time scale of ion motions, demonstrated an increased slope in the vicinity of the liquid–liquid transition, originating from the self-organization of trihexyl(tetradecyl)phosphonium cations,^26,27^ as evidenced by SAXS measurements. The same behavior has also been observed for the temperature evolution of the viscosity (η) of [P_666,14_][DCA] and the ion dynamics of other ILs with evidence of liquid–liquid transition.^28,29^

A significant advantage of the BDS technique is the possibility of studying the dynamics of ions and, thus, their nanoordering at elevated pressure.^30^ It has been many times demonstrated that the dc-conductivity (σ_dc_), quantifying the ion dynamics of protic, aprotic, and polymerized ILs, can be determined with very high precision at various *T*–*P* thermodynamic conditions, not only in the equilibrium liquid state but also near the liquid–glass transition.^31,32^ This gives a unique opportunity to investigate the effect of the pressure on the self-assembly of ILs. An additional benefit of high-pressure experiments is that in contrast to isobaric cooling during isothermal squeezing, the thermal energy of ions remains constant. Consequently, the effects of temperature and density on IL self-assembly can be separated.

Here, we investigate the temperature and pressure behavior of ionic conductivity in trihexyl-(alkyl)phosphonium-based ILs, [P_666,*n*_][TFSI] (*n* = 2, 6, 8, 12). According to recent studies, all these materials can be easily supercooled and reveal a tendency to self-assembly, as evidenced by aliphatic chain coupling due to their mutual ordering.^33^ Therefore, they are good candidates for studying the effects of pressure on the nanoorganization of ILs. We found that self-assembly of cation alkyl chains taking place in [P_666,*n*_][TFSI] ILs affects the rate of ion dynamics both at ambient and elevated pressures, leading to the peculiar behavior of σ_dc_(*T*^–1^) near the liquid–glass transition and the inflection point at high pressure.

## Experimental Methods

### Materials

**Figure 1:**
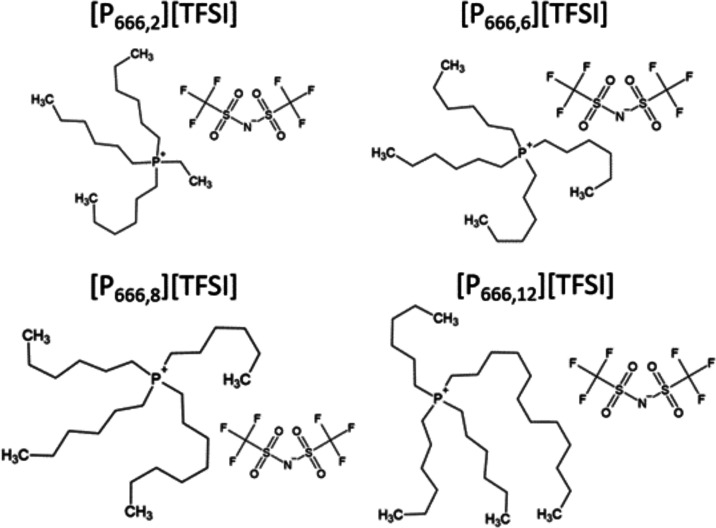
Chemical structures of the studied ILs.

The tested samples are trihexyl-alkylphosphonium bis(trifluoromethylsulfonyl)imide [P_666,*n*_][TFSI] (*n* = 2, 6, 8, 12) samples, which have different alkyl chain lengths in the cation. These samples were synthesized and purified in the QUILL Centre with a purity of more than 98%, which was reported in our previous work.^33^ All studied ILs are colorless and transparent liquids at room temperature, and we used samples without further treatment. The chemical structures of the studied ILs are presented in Figure 1.

### Broadband Dielectric Spectroscopy

The electrical conductivity measurements of the investigated ILs at ambient pressure were conducted by using a Novo-Control GMBH Alpha dielectric spectrometer, covering a wide range of temperatures and frequencies (from 10^–2^ to 10^7^ Hz). The Novocool system was used to control the temperature precisely with an accuracy of 0.1 K during the experiments. Simultaneously, the sample was securely positioned between two stainless-steel electrodes, each with a diameter of 15 mm, and a 0.1 mm quartz ring, ensuring stable electrode separation.

The capacitor hermetically closed in a Teflon capsule was used for high-pressure dielectric measurements. This capsule was placed in a high-pressure chamber. The sample compression was achieved by using silicone oil. Note that the sample was not in contact with the oil. The pressure was accurately monitored by a Unipress setup with a resolution of 1 MPa, while temperature regulation was maintained within 0.1 K and facilitated by a Weiss refrigerator.

### Raman Measurements

Temperature-dependent Raman spectroscopy was conducted using a WITec confocal Raman microscope alpha 300R, equipped with a solid-state laser (λ = 457 nm). The laser was integrated into the microscope via a polarization-maintaining single-mode optical fiber with a diameter of 50 μm. The laser beam was focused on the sample through a long-distance Olympus MPLAN objective (50×/0.76NA), while the scattered light was collected through a multimode fiber with the same diameter. Before measurements, the spectrometer’s monochromator, featuring a 600 lines/mm grating, was calibrated using a silicon reference plate (520.7 cm^–1^). For the experiments, samples were placed as minute droplets on cover glasses and positioned on a THMS600 Linkam stage. Room-temperature Raman spectra of [P_666,*n*_][TFSI] for *n* = 2, 6, 8, 12 were acquired with a laser power of 20 mW directed at the sample utilizing 10 scans, an integration time of 20 s, and a spectral resolution of 3 cm^–1^. Subsequently, each sample was progressively cooled to 223 K in steps of 10 K and further down to 173 K in steps of 2 K, maintaining a cooling rate of 10 K/min and a temperature stabilization accuracy of 0.1 K. At each temperature point, Raman spectra were collected with ten scans, an integration time of 10 s, and a resolution of 3 cm^–1^. All acquired spectra underwent postprocessing, including cosmic ray removal and baseline correction utilizing WITec Project Five Plus software (5.1.1). Finally, the Raman spectra were normalized to elucidate the temperature-dependent changes more clearly.^34^

## Results and Discussion

### Ambient Pressure Studies of Ion Dynamics

**Figure 2:**
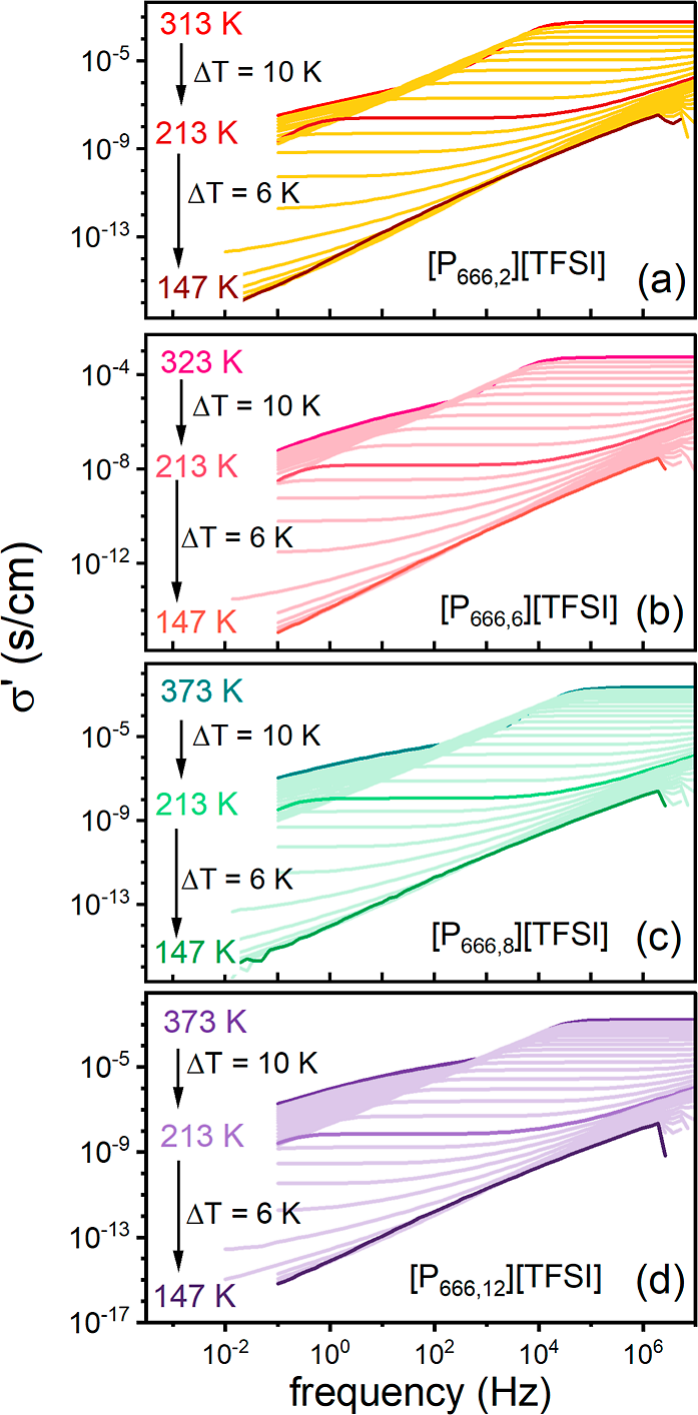
Real part of complex electric conductivity as a function of frequency for (a) [P_666,2_][TFSI], (b) [P_666,6_][TFSI], (c) [P_666,8_][TFSI], and (d) [P_666,12_][TFSI].

It is well-known that most ILs are good glass formers, and the same applies to the samples examined here. We recently found that all tested [P_666,*n*_][TFSI] can be easily supercooled, and cold crystallization occurs only on slow heating from the glassy state.^33^ Herein, the dielectric measurements of [P_666,*n*_][TFSI] were performed from the temperatures covering the normal liquid through the supercooled liquid to the glass phase. The obtained dielectric data are presented in the conductivity formalism in Figure 2. As can be seen, the real part of complex electric conductivity σ′(*f*) measured over a wide frequency range (10^–2^ to 10^7^ Hz) has three well-defined regions: (i) a frequency-independent dc-conductivity (σ_dc_) seen as a plateau, (ii) a power-law behavior on the high-frequency side that is slightly affected by secondary relaxation related to intramolecular motions of long alkyl chains of cation, and (iii) a left-side decrease of σ′ from σ_dc_ denoting the electrode polarization effect. It appears that the dc-conductivity plateau shifts to a lower frequency as temperature decreases, indicating that temperature is an essential factor affecting the conductivity of ILs.

**Figure 3:**
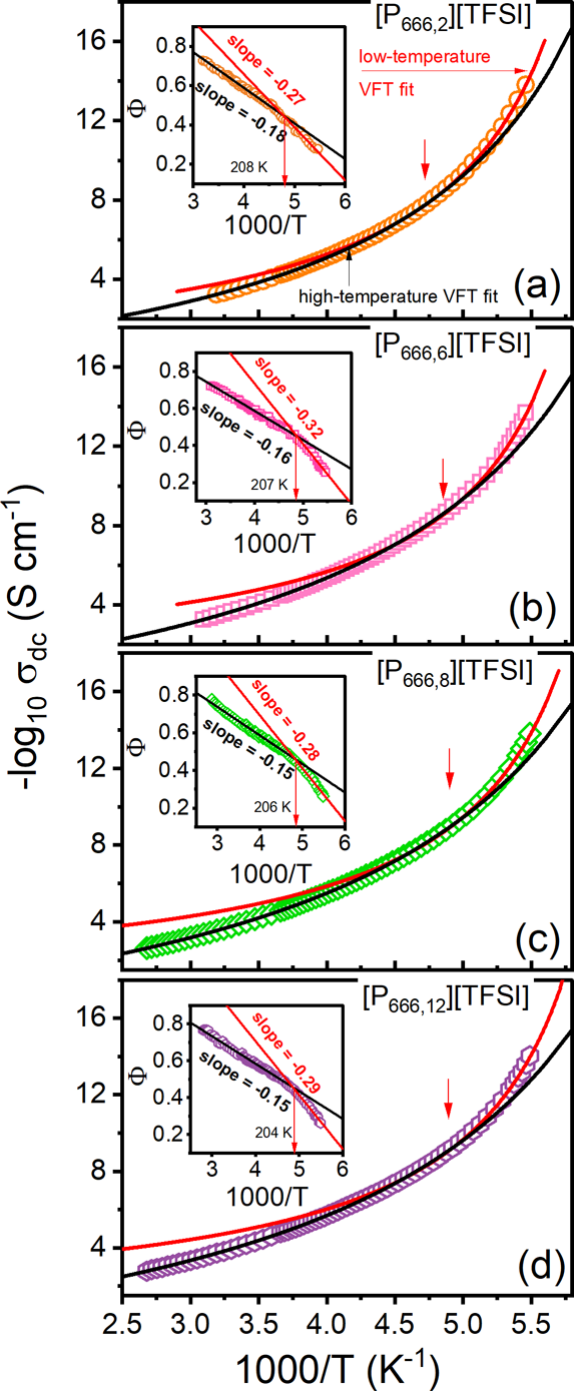
Temperature dependence of dc-conductivity for the studied ILs. The solid lines denote the fits to the VFT equation (eq 1). The insets present Stickel plots.

Since the frequency-dependent dielectric spectra of [P_666,*n*_][TFSI] display quasi-universal behavior, the temperature evolution of σ_dc_ must be examined to describe ion dynamics in more detail. The values of σ_dc_ were determined directly from the frequency-independent part of σ′(*f*). As depicted in Figure 3, the dc-conductivity of [P_666,*n*_][TFSI] shows the characteristic non-Arrhenius temperature dependence commonly observed in glass-forming systems. Therefore, according to standard practice, the empirical Vogel–Fulcher–Tammann (VFT) equation^35^ (eq 1) has been initially used to parameterize the experimental data
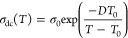
1where σ_0_ denotes the pre-exponential factor, *D* is the strength parameter characterizing the deviation from Arrhenius behavior, and *T*_0_ denotes the Vogel temperature (sometimes *T*_0_ is also called an ideal glass transition temperature). However, in contrast to other aprotic ILs investigated in the literature,^36^ a single VFT function is not enough to describe the dc-conductivity behavior over the entire temperature range. Therefore, two VFT functions have been used to parameterize the 12 decades of σ_dc_. The obtained fitting parameters, together with the fragility index *m* calculated from the strength parameter *D* via *m* = 16 + 590/*D*,^37^ are listed in Table 1. As can be seen in Figure 3, in the vicinity of the liquid–glass transition temperature (defined as σ_dc_ = 10^–14^ S cm^–1^),^38^ the experimental data are more sensitive to temperature changes than expected from the high-temperature VFT fit. In other words, at a specific temperature, around 18–24 K higher than *T*_g_ (*T* = 204–208 K, depending on the cation alkyl chain length), the ion dynamics of [P_666,*n*_][TFSI] ILs slows down substantially. On the other hand, the low-temperature VFT function interpolates 8 decades of data properly, while the rest of the points fall below the fit. At first sight, in Figure 3, it is also visible that high- and low-temperature VFT functions differ substantially in fragility. While the former can be classified as strong, the latter is evidently more fragile. Indeed, the parameters collected in Table 1 show a 2-fold increase in fragility between high- and low-temperature dynamics.

**Table 1:** VFT Parameters Obtained from Fitting of dc-Conductivity for the Studied ILs

	high-temperature VFT				low-temperature VFT				
ILs acronym	log σ_0_ (S/cm)	*D*	*T*_0_ (K)	*m*	log σ_0_ (S/cm)	*D*	*T*_0_ (K)	*m*	*T*_g_ (K)
[P_666,2_][TFSI]	–0.04	9.59	138.1	77	1.48	5.47	153.5	124	182.7
[P_666,6_][TFSI]	–0.19	11.75	130.3	66	2.38	4.62	155.4	144	182.2
[P_666,8_][TFSI]	–0.21	12.64	127.5	62	2.49	4.94	152.9	135	181.4
[P_666,12_][TFSI]	–0.15	13.31	125.7	60	2.58	5.05	152.8	133	182.1

To explain these results, two scenarios can be taken into account. The first one accounts for the existence of some intermediate temperature, called *T*_b_, commonly identified with the onset of complex dynamics.^39−42^ Specifically, for many glass-forming liquids, *T*_b_ separates the temperature dependence of relaxation dynamics into two regions with high-temperature simple dynamics at *T* > *T*_b_ and low-temperature complex dynamics at *T* < *T*_b_, while both are characterized by different VFT behaviors. However, one should note that *T*_b_, identified as the onset of cooperativity, is usually observed at a relaxation rate of about 10^–9^ s that corresponds to σ_dc_ in the order of 10^–3^ S/cm,^43,44^ while the departure of low-temperature VFT from experimental data occurs at dc-conductivity around three decades lower for [P_666,*n*_][TFSI]. Furthermore, the *T*_b_ ≈ 1.2....1.3 × *T*_g_ rule also does not hold for examined ILs. In this context, an alternative explanation that takes into account the changes in IL self-organization needs to be considered. In this context, a significant deviation from a single VFT law recently observed for several phosphonium ILs at the temperature of liquid–liquid transition, *T*_LL_,^26,29^ needs to be recalled. Namely, it has been shown that tetra(alkyl)phosphonium ILs with a 14-carbon alkyl chain exist in two supercooled phases differing from each other in a tendency toward self-assembly and temperature sensitivity of ion dynamics. Specifically, the conventional liquid was classified as strong, while the self-assembled supercooled state with apolar domains formed by long alkyl chains of the cation revealed a fragile nature. Furthermore, the derivative analysis of experimental τ_σ_(*T*^–1^) data, [*d*(log τ_σ_)/*d*(1000/*T*)]^−0.5^, showed a substantial deviation from the straight line in the direction opposite to that found for other glass-forming liquids^45^ with characteristic *T*_b_ temperature.^29^ To check if the same is true of the materials tested herein, the Stickel operator, which linearizes the VFT law, has been employed for σ_dc_(*T*^–1^) data, Φ = [*d*(−log σ_dc_)/*d*(1000/*T*)]^−0.5^.^46−48^ As presented in the insets of Figure 3, the Stickel plot of examined samples reveals two linear regions that intersect at some intermediate temperature (*T*_cross_), being slightly different between the examined ILs (208 → 204 K from [P_666,2_] to [P_666,12_]). Moreover, the slope of the Φ(1000/*T*) dependence doubles when we move from a high-to low-temperature region. An exception is [P_666,2_][TFSI], for which only a slight increase in slope occurs. Herein, one should note that the Stickel behavior observed for [P_666,*n*_][TFSI] ILs is not as pronounced as for [P_666,14_]-based ILs with clear evidence of liquid–liquid phase transition.^29^ However, this could be expected since the LLT did not show up on the DSC thermograms of [P_666,*n*_][TFSI] ILs.^33^ To recognize the structural origin of changes in ion dynamics, temperature-dependent Raman measurements were performed on all examined ILs herein.

### Identification of Structural Changes Accompanying Peculiarities in Ion Dynamics

**Figure 4:**
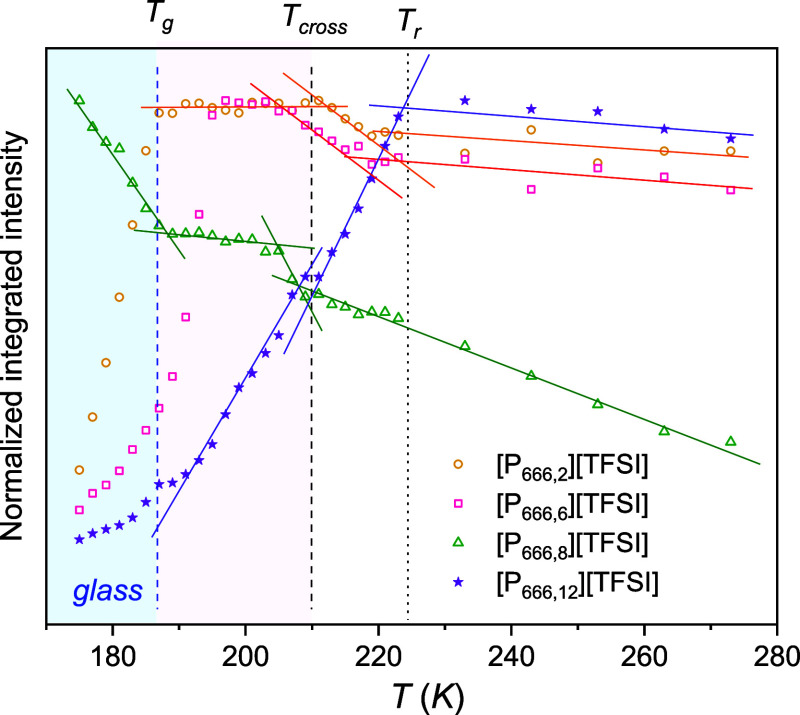
Temperature-dependent behavior of normalized integrated intensity of the ν_as_CH_2_ band around 2880 cm^–1^ with highlighted characteristic temperatures, glass temperature (*T*_g_), cross temperature (*T*_cross_) and reorganization temperature (*T*_r_).

Temperature-dependent Raman spectroscopy was employed to examine the effect of the aliphatic chain length on the tendency of the [P_666,*n*_][TFSI] ILs for self-organization in a broad temperature range (273–173 K). The recorded spectra are presented in Figure S1, while Figure 4 shows the temperature-dependent integrated intensity analysis for a band related to the asymmetric stretching vibration of methyl and methylene groups. As can be seen, a nonmonotonic pattern was observed for all examined ILs. The first change in the slope identified around 224 K indicates a molecular reorganization associated with the alignment of the alkyl chains. Beyond the second change, taking place around the crossover temperature (*T*_cross_) determined from BDS measurements, there was an alteration in integrated intensity values, pointing to a notable reduction in molecular mobility (Figure 4). As a result, the structural reorganization of cations is, to some extent, frozen below *T*_cross_.

**Figure 5:**
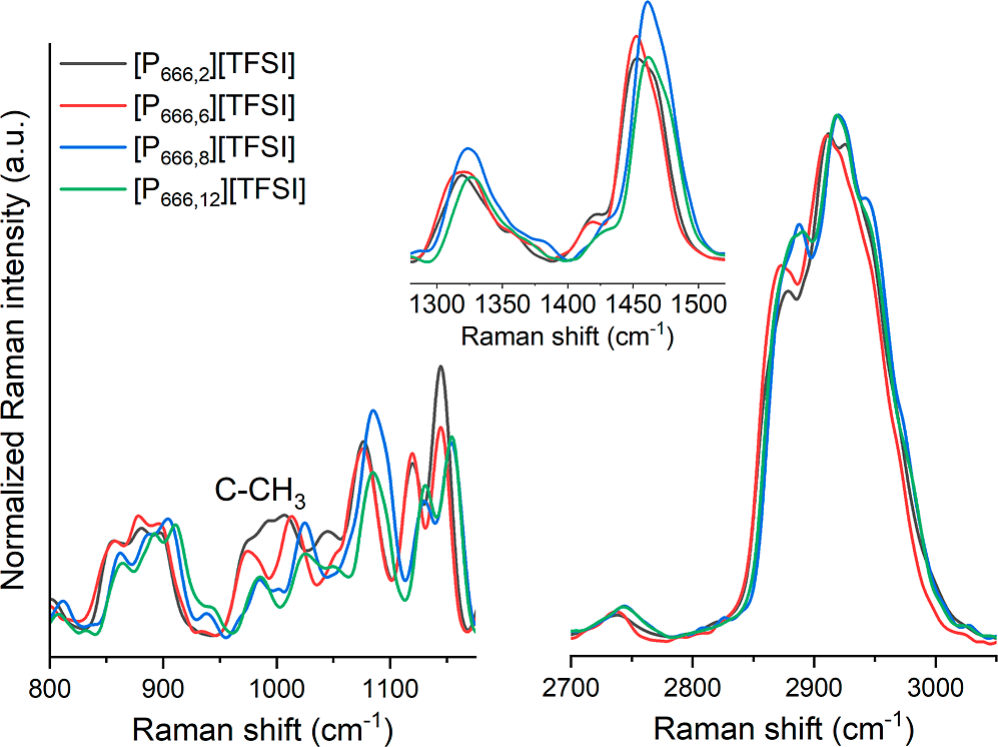
Raman spectra of [P_666,*n*_][TFSI] obtained at *T* = 197 K. Data are summarized in the three spectral ranges: 800–1180, 1280–1520, and 2700–3050 cm^–1^.

A closer examination of the spectra recorded below the *T*_cross_ value highlighted significant band configurations in their intensity and positions. These differences were particularly pronounced when analyzing the spectral data of [P_666,*n*_] ILs with shorter aliphatic chains (*x* ≤ 6) compared to those with a higher number of methylene groups (*x* > 6). Additionally, a clear preference for the gauche state in the examined systems was manifested by bands at 1078 and 1120 cm^–1^ for chains shorter in length and at 1085 and 1130 cm^–1^ for longer chains (see Figure 5). For [P_666,8_]^+^ and [P_666,12_]^+^ systems, a notable shift toward higher frequencies was observed, indicating an increased tendency for aliphatic chain segregation and deceleration of molecular dynamics, as documented by Harris et al.^27^ This trend was similarly observed for bands characteristic of straight-chain alkanes with stretching ν(C–C–C) at 859, 885, and 898 cm^–1^; deformational δ(CH_3_) and δ(CH_2_) bands around 1460 cm^–1^ for [P_666,8_]^+^ and [P_666,12_]^+^; and stretching δ(CH_3_) and δ(CH_2_) bands at 2886 and 2920 cm^–1^, highlighting an upward shift of approximately 6–10 cm^–1^ compared to those of [P_666,2_]^+^ and [P_666,6_]^+^ systems. Lowering the temperature to 191 K proved the further reorganization of aliphatic chains in [P_666,6_]^+^ toward the molecular geometry observed for [P_666,8_]^+^ and [P_666,12_]^+^ systems.

Based on the described Raman measurements performed over a wide temperature range, one can claim that the change in conductivity behavior from strong to fragile observed for [P_666,*n*_][TFSI] ILs is due to the self-organization of cations that takes place at *T*_cross_ ≈ 1.1*T*_g_. In this context, the pressure evolution of dc-conductivity is fascinating and will be addressed below.

### High-Pressure Studies of Ion Dynamics

As is well-known, isothermal compression influences ion dynamics only by reducing the free volume. Therefore, it is a valuable tool to understand the ion transport of self-assembled liquids. To address this issue, we performed isothermal dielectric measurements for [P_666,*n*_][TFSI] (*n* = 2, 8, 12) within the temperature range of 204–242 K, i.e., where the onset of cation self-organization was detected at ambient conditions. Since applying pressure strongly enhanced the tendency of [P_666,6_][TFSI] to crystallize, it has been excluded from high-pressure studies. A high crystallization tendency upon compression of [P_666,6_][TFSI] can be due to the symmetric structure of the cation combined with variations in the ordering of the alkyl chains found in Raman measurements.

**Figure 6:**
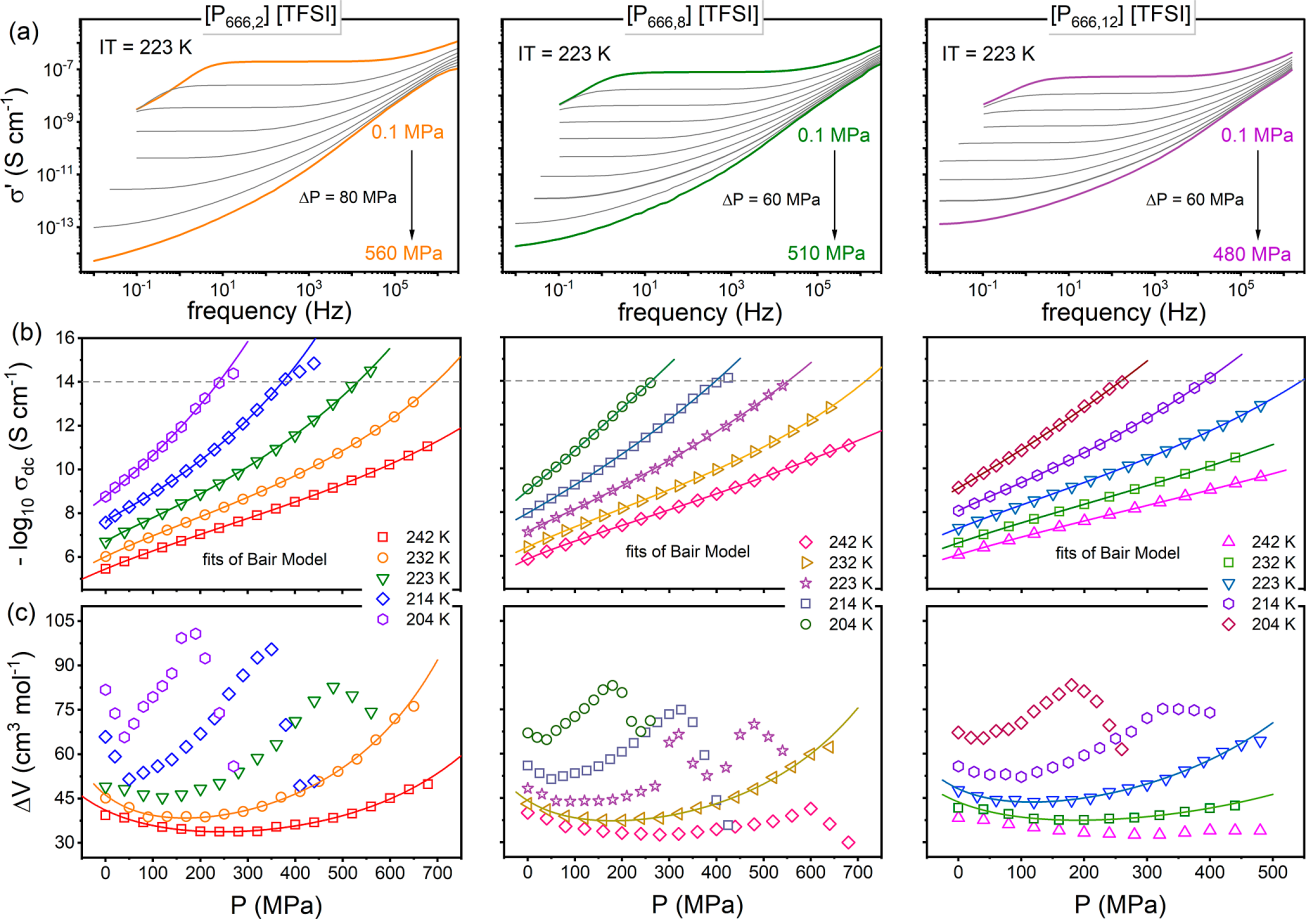
(a) Representative dielectric spectra for [P_666,2_][TFSI], [P_666,8_][TFSI], and [P_666,12_][TFSI] obtained at 223 K. Panel (b) presents the pressure dependence of log_10_σ_dc_ for [P_666,2_][TFSI], [P_666,8_][TFSI], and [P_666,12_][TFSI] at various temperatures. The solid lines are fits of the Bair model (eq 3) to experimental data. Panel (c) shows their activation volume as a function of pressure at the same temperatures. The lines denote the fits according to eq 3.

**Figure 7:**
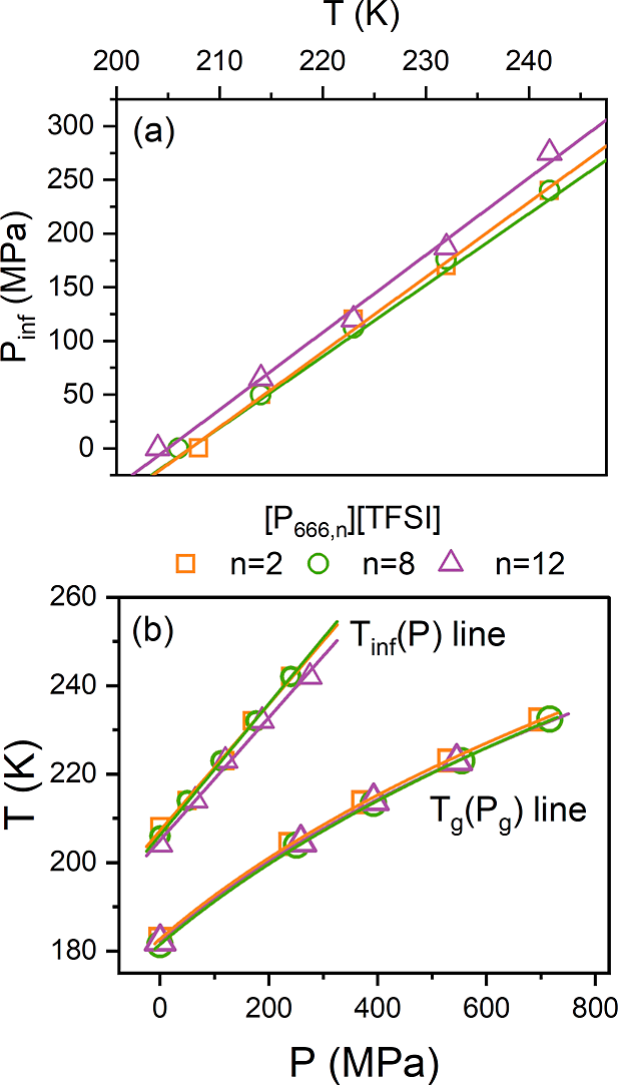
(a) Temperature dependence of the inflection pressure for [P_666,2_][TFSI], [P_666,8_][TFSI], and [P_666,12_][TFSI]. (b) Pressure dependence of the glass transition temperature and an inflection point for [P_666,2_][TFSI], [P_666,8_][TFSI], and [P_666,12_][TFSI] with the fits of the Andersson–Andersson equation (solid lines).

Figure 6a illustrates the effect of pressure on the dielectric response of [P_666,2_][TFSI], [P_666,8_][TFSI], and [P_666,12_][TFSI] at 223 K. As presented, increasing pressure exerts a similar effect on dielectric spectra as decreasing temperature, i.e., the σ_dc_ plateau appears at lower values and moves toward lower frequencies during squeezing. To quantify the former effect, −log_10_ σ_dc_ as a function of pressure has been determined and presented in Figure 6b. At first sight, the isothermal dependences of all examined ILs follow conventional nonlinear behavior, with a kink observed at 10^–14^ S cm^–1^ for a few of them, identifying liquid–glass transition pressure (*P*_g_). However, the analysis of the data in terms of the activation volume revealed a unique feature of log_10_σ_dc_(*P*) experimental data. Specifically, activation volume Δ*V* was calculated by using the following formula^49^
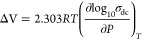
2where *R* is the gas constant, shows a distinct minimum (see Figure 6c). Such a phenomenon, called an inflection point, is a consequence of the concave–convex character of σ_dc_(*P*) dependences visible after closer inspection. Therefore, for proper parameterization of pressure dependences of dc-conductivity for [P_666,*n*_][TFSI] ILs, the phenomenological model proposed by Bair^50^ (eq 3) instead of the typical pressure counterpart of the VFT equation (pVFT),^51^, has been used
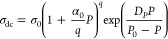
3where α_0_ and *q* denote the McEwen parameter and exponent, respectively. Note that, in fact, the Bair model combines the pVFT law (for faster-than-exponential) with the McEwen equation () (for a slower-than-exponential response), which was initially employed to characterize the pressure dependence of viscosity. As can be seen in Figure 6b, the fits of this hybrid model to σ_dc_(*P*) data of [P_666,*n*_][TFSI] follow the experimental points perfectly and thus properly interpolate the Δ*V*(*P*) dependences. From a closer inspection of Figure 6c, it can be seen that the inflection point shifts to higher pressures as the temperature increases. To quantify this effect, in the next step, we have defined *P*_inf_ as the minimum in isothermal Δ*V*(*P*) data and plotted it as a function of temperature in Figure 7a. For all studied ILs, *P*_inf_(*T*) follows the linear behavior, with the slope being sample-dependent. Furthermore, an extrapolation to ambient conditions corresponds well with the temperature at which the characteristic crossover in the Stickel plot has been observed. The σ_dc_ determined at *T*_cross_ and σ_dc_(*P*_inf_) are also in the same order of magnitude. These results legitimate us to conclude that the inflection point characterizing the pressure dependence of σ_dc_ in [P_666,*n*_][TFSI] can be considered an inherent feature of ion dynamics governed by structural self-assembly.

Interestingly, over the years, only a limited number of glass-forming materials have been proven to reveal inflection points experimentally—that is, glycerol, propylene carbonate (PC),^**Error! Bookmark not defined**,^^52^ 2-butyl-1-octanol,^30^ protic IL [C_8_HIm][NTf_2_],^53^ and recently [P_666,14_][BOB].^54^ Casalini and Bair suggested that the origin of the inflection point in PC can be attributed to the pressure dependences of compressibility and the apparent activation energy at a constant volume. However, it has also been speculated that PC behaves differently than other van der Waals liquids due to its tendency to dimerize, which could lead to the formation of some local structures.^55^ At the same time, glycerol and [C_8_HIm][NTf_2_] generate strong hydrogen bonds and are therefore expected to form aggregates. In turn, intensified molecular clustering of some associating liquids has been found at elevated pressure.^56^ The last example, [P_666,14_][BOB], has undergone a hidden liquid–liquid transition originating from the self-organization of alkyl cation chains in nonpolar domains. All these examples suggest that the inflection point can be associated with structural self-assembly.

As a final point, a charge transport mechanism through the self-assembled structures will be examined. Specifically, we will check whether the examined ILs behave like conventional aprotic ILs with charge transport fully controlled by viscosity or if they mimic single-ion conductors with charge transport decoupled from structural dynamics. To verify which scenario is true for [P_666,*n*_][TFSI], the dc-conductivity at the liquid–glass transition temperature and pressure needs to be examined. According to literature data, σ_dc_ in the order of 10^–14^ S cm^–1^ at *T*_g_ (or *P*_g_) characterize aprotic, protic, and polymerized ILs with vehicle-type conduction. On the other hand, σ_dc_ larger than this value indicates the existence of fast charge transport to some extent, independent of structural dynamics.^57,58^ As seen in Figures 3 and 6b, σ_dc_(*T*^–1^) and σ_dc_(*P*) data follow the fitting curve (VFT and hybrid model, respectively) up to 10^–14^ S cm^–1^. After that, a crossover to linear dependence, a manifestation of the liquid–glass transition, is observed for high-pressure data. Therefore, for examined ILs, *T*_g_ (and *P*_g_) can be defined in a classical way as σ_dc_ = 10^–14^ S cm^–1^. Consequently, despite the self-organization of cationic species, charge transport in [P_666,*n*_][TFSI] remains controlled by viscosity. Since within the available pressure range, only a few isotherms of [P_666,*n*_][TFSI] reach the time scale of 10^–14^ S cm^–1^, simple extrapolation of isothermal σ_dc_(*P*) data by the hybrid Bair model needs to be employed to determine the liquid–glass transition pressure *P*_g_ at higher temperatures. As a result, the *T*_g_(*P*_g_) dependence for [P_666,2_][TFSI], [P_666,8_][TFSI], and [P_666,12_][TFSI] was determined and is plotted in Figure 7b. As can be seen, all examined ILs reveal nearly identical nonlinear pressure behavior of *T*_g_. Therefore, it is not surprising that the parameterization of *T*_g_(*P*_g_) data follows the empirical Andersson–Andersson equation.  gives the exact value of d*T*_g_/d*P* coefficient (*k*_1_/*k*_3_) equal to 105 ± 1 K/GPa for all tested systems. This result indicates that the pressure sensitivity of the ion dynamics remains constant with increasing alkyl chain length in the case of [P_666,*n*_]-based ILs. For the completeness of our analysis, the pressure coefficient for the inflection point d*T*_inf_/d*P* was also determined. For this purpose, the data presented in Figure 5a have been visualized in a pressure representation in Figure 5b. As can be seen, in contrast to the *T*_g_(*P*) dependences, *T*_inf_(*P*) follows the linear behavior for all examined ILs. Furthermore, it is much steeper, which is reflected in higher d*T*_inf_/d*P*. The values of d*T*_inf_/d*P* determined in the limit of ambient pressure are equal to 143, 148, and 139 K/GPa for [P_666,2_][TFSI], [P_666,8_][TFSI], and [P_666,12_][TFSI], respectively, and again lead to the conclusion that the pressure behavior of ion dynamics does not depend on the cation alkyl chain length.

## Conclusions

Here, using BDS, we investigated the conductivity behavior for a series of aprotic ILs composed of a hydrophobic bis(trifluoromethanesulfonyl)imide anion ([TFSI]^−^) and tetra(alkyl)phosphonium cations ([P_666,*n*_]^+^, *n* = 2, 6, 8, 12) at ambient and elevated pressure. For all examined ILs, the temperature dependence of the dc-conductivity exhibits non-Arrhenius behavior, typical for conventional glass-forming liquids. However, the derivative analysis of σ_dc_(*T*^–1^) dependences shows two regions with different ion dynamics. The first high-temperature one characterizes the conventional supercooled state, while the second, with the onset at *T*_cross_ ≈ 1.1*T*_g_, refers to the supercooled state influenced by local changes in ion–ion interaction and organization of cation alkyl chains. The high-pressure isothermal measurements performed in the temperature range of ionic self-assembly revealed a unique concave–convex nature of σ_dc_(*P*) dependences and, thus, a so-called inflection point in pressure dependence of activation volume Δ*V*(*P*). Consequently, one might conclude that the tendency of an aliphatic chain to segregate in [P_666,*n*_][TFSI] ILs affects the rate of ion dynamics, leading to the inflection point at elevated pressure.

## Supporting Information Available

Raman measurements of the examined ILs (PDF)
