# Supplementary material for: Inflection Point in Pressure Dependence of Ionic Conductivity as a Fingerprint of Local Structure Formation
Source: J Phys Chem B. 2024 May 8;128(20):5109–17. doi: 10.1021/acs.jpcb.3c08506 (PMC11129299; doi:10.1021/acs.jpcb.3c08506)
Supplement: Supplementary file 1 — jp3c08506_si_001.pdf [file jp3c08506_si_001.pdf]

Supporting Information

The Inflection Point in Pressure Dependence of Ionic Conductivity as a Fingerprint of Local Structure Formation

S. Koymeth[1], B. Yao[1], M. Paluch[1], M. Dulski[2], M. Swadzba-Kwasny[3], Z. Wojnarowska[1*]

[1] *Faculty of Science and Technology, Institute of Physics, University of Silesia in Katowice, 75 Pułku Piechoty 1A, 41−500 Chorzów, Poland*

[2] *Faculty of Science and Technology, Institute of Materials Science, the University of Silesia in Katowice, 75 Pułku Piechoty 1A, 41−500 Chorzów, Poland;*

[2]*The QUILL Research Centre, School of Chemistry and Chemical Engineering, The Queen's University of Belfast, David Keir Building, Stranmillis Rd, BT9 5AG Belfast, NI, UK.*

Raman measurements of examined ILs

The bands corresponding to vibrational modes of the aliphatic chains of the cation $[P_{666,n}]^+$, including straight-chain alkanes (800 – 1175 cm^{-1}) coupled with methyl-anchored unsaturated C-C skeletal vibrations (900 – 1130 cm^{-1}), deformational modes of CH_x (x = 2,3) (1275 - 1525 cm^{-1}) coupled with methyl-anchored unsaturated C-C skeletal vibrations (1355 – 1480 cm^{-1}), were examined (see Fig.1S). All those bands overlap with the CH_x (x = 2,3) wagging, rocking, and twisting vibrations of aliphatic chains (715 – 1430 cm^{-1}). Finally, the most intense bands are the symmetric and asymmetric stretching vibration of CH_x (x = 2,3) (2800 - 3050 cm^{-1}). Notably, the fingerprint region's spectral pattern explains the conformational order and degree of coupling.

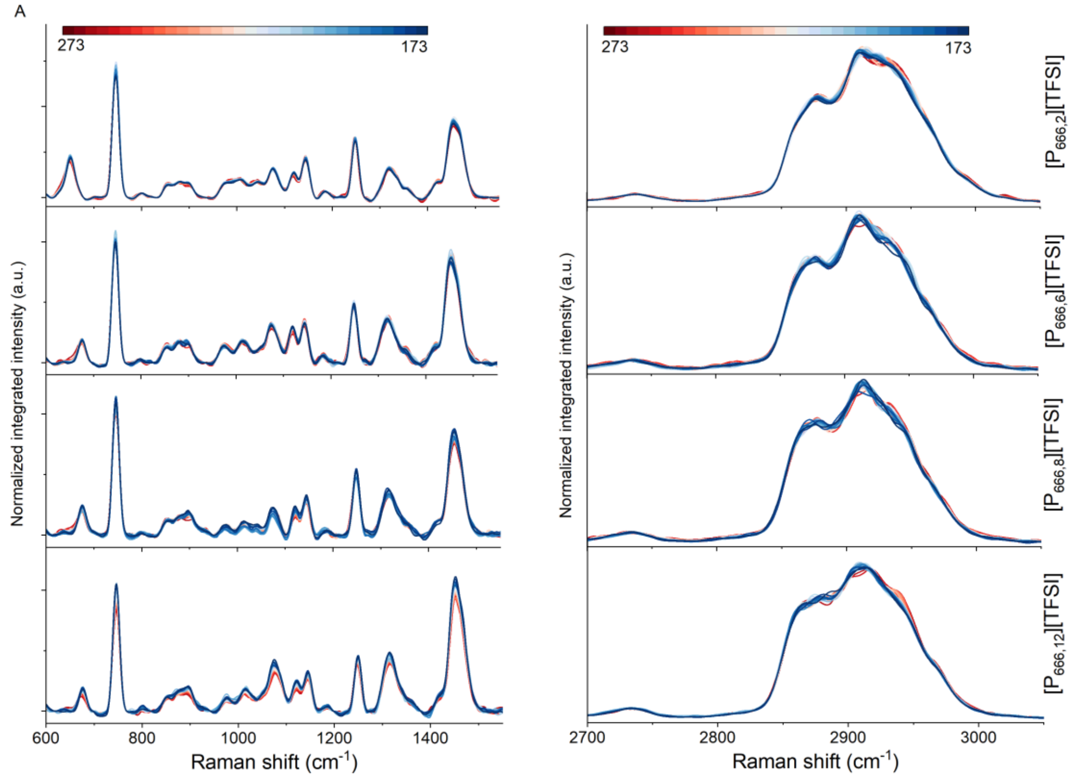

Fig. S1. Temperature-dependent Raman spectra of $[P_{666,n}][TFSI]$ measured in a cooling regime from 273 down to 173 K. Data were summarized in the two spectral ranges: 600 - 1550 cm^{-1} range (left panels) and 2700 - 3050 cm^{-1} (right panels).
